# Explainable Machine Learning for Real-Time Hypoglycemia and Hyperglycemia Prediction and Personalized Control Recommendations

**DOI:** 10.1177/19322968221103561

**Published:** 2022-06-13

**Authors:** Christopher Duckworth, Matthew J. Guy, Anitha Kumaran, Aisling Ann O’Kane, Amid Ayobi, Adriane Chapman, Paul Marshall, Michael Boniface

**Affiliations:** 1 Electronics and Computer Science, IT Innovation Centre, University of Southampton, Southampton, UK; 2 Department of Medical Physics, University Hospital Southampton NHS Foundation Trust, Southampton, UK; 3 Child Health, Department of Endocrinology, University Hospital Southampton NHS Foundation Trust, Southampton, UK; 4 Human-Computer Interaction for Health, University of Bristol, Bristol, UK; 5 UCL Interaction Centre, University College London, London, UK; 6 Electronics and Computer Science, University of Southampton, Southampton, UK

**Keywords:** continuous glucose monitoring, explainable and trustworthy AI, feature extraction, hypoglycemia prediction, hyperglycemia prediction, machine learning

## Abstract

**Background:**

The occurrences of acute complications arising from hypoglycemia and hyperglycemia peak as young adults with type 1 diabetes (T1D) take control of their own care. Continuous glucose monitoring (CGM) devices provide real-time glucose readings enabling users to manage their control proactively. Machine learning algorithms can use CGM data to make ahead-of-time risk predictions and provide insight into an individual’s longer term control.

**Methods:**

We introduce explainable machine learning to make predictions of hypoglycemia (<70 mg/dL) and hyperglycemia (>270 mg/dL) up to 60 minutes ahead of time. We train our models using CGM data from 153 people living with T1D in the CITY (CGM Intervention in Teens and Young Adults With Type 1 Diabetes)survey totaling more than 28 000 days of usage, which we summarize into (short-term, medium-term, and long-term) glucose control features along with demographic information. We use machine learning explanations (SHAP [SHapley Additive exPlanations]) to identify which features have been most important in predicting risk per user.

**Results:**

Machine learning models (XGBoost) show excellent performance at predicting hypoglycemia (area under the receiver operating curve [AUROC]: 0.998, average precision: 0.953) and hyperglycemia (AUROC: 0.989, average precision: 0.931) in comparison with a baseline heuristic and logistic regression model.

**Conclusions:**

Maximizing model performance for glucose risk prediction and management is crucial to reduce the burden of alarm fatigue on CGM users. Machine learning enables more precise and timely predictions in comparison with baseline models. SHAP helps identify what about a CGM user’s glucose control has led to predictions of risk which can be used to reduce their long-term risk of complications.

## Introduction

People with type 1 diabetes (T1D) face a daily balance to keep their glucose levels within safe levels (ie, “in-range”). Severe complications are prevalent and arise from glycemic variability, low blood sugars (hypoglycemia), and high blood sugars (hyperglycemia).^1^ For hypoglycemic incidents alone, the requirement for emergency assistance may be as high as 7.1% per year^2^ and could account for 6% to 10% of deaths for those with T1D.^3,4^ Long-term impacts of hypoglycemia include impacts on cognition and potential links with dementia.^5^ In addition, frequent hyperglycemia can lead to short-term risk such as diabetic ketoacidosis and long-term complications such as retinopathy, neuropathy, nephropathy, and cardiovascular disease.^6-8^ Effective glucose management for adolescents and young adults living with T1D is challenging,^9,10^ due to the multiple transitions taking place in their lives, including puberty, relationships, the move to more independent living, and diabetes self-care, and also the transfer from pediatric to adult clinical care teams. Parental fear of severe complications is prevalent throughout these transitional years.^11-13^

Continuous glucose monitoring (CGM) enables regular automated readings of estimated glucose levels found in interstitial fluid, providing immediate insight into glucose control. Continuous glucose monitoring has been demonstrated to reduce the risk of both hypoglycemia and hyperglycemia, along with reducing daily glycemic variability for users with T1D.^14-16^ In addition to mitigating short-term risk of severe hypoglycemia and hyperglycemia, compliance of wearing CGM devices has been shown to improve glycosylated hemoglobin (HbA1c) levels, which, if sustained, reduce long-term complication risks.^17,18^ The magnitude of reduction in HbA1c from CGM usage is dependent on the user’s original HbA1c value, ie, those at highest risk of complications from poorer control are likely to benefit the most.^16^ Specific to young adults, Laffel et al^19^ demonstrate a clear improvement in HbA1c for those utilizing CGM.

Real-time CGM devices provide alerts for users when their interstitial fluid glucose falls above or below a desired range. Type 1 diabetes management can be aided further by having *ahead-of-time* predictions so individuals can identify risk early and better plan self-care activities, such as insulin dosages. Simple threshold-based algorithms have been able to successfully predict hypoglycemia 30 minutes in advance (eg, Medtronic-640 “SmartGuard”^20^). More complex statistical models and machine learning algorithms enable more accurate prediction and are able to extend this prediction horizon.^21-28^ Dave et al^23^ emphasize the importance of feature extraction when generating predictions of hypoglycemia in CGM data. Generating features that are both predictive in models and insightful for understanding a user’s glucose control is a difficult balance.

In this work, we make two novel contributions: algorithms tailored to young adults and explanations. First, we introduce machine learning models to predict hypoglycemia (<70 mg/dL) and hyperglycemia (>270 mg/dL)^29^ with a trustworthy prediction horizon up to 60 minutes (44 minutes on average) for young adult users of CGM. While CGM risk prediction is a well-explored topic, more must be done to understand what led to increased risk for an individual so they can be proactive. We introduce using *explainable* machine learning, to not only predict risk, but to automatically identify the most important factors in an individual’s CGM data that led to increased risk. Explanations have no detrimental impact on model performance. We provide a framework in which machine learning can be used to:

1. Provide real-time predictions of hypoglycemia and hyperglycemia (Results—Model Evaluation) using intuitive features (Methods—Features) generated from CGM data (Methods—Data).
2. Automatically identify the most important features that have led to predictions of risk for each CGM user over a given time period (Results—Model Explanation).
3. Provide personalized control recommendations for each CGM user to help with their T1D management (Results—User Interface).

## Methods

### Data

We make use of publicly available data from “A Randomized Clinical Trial to Assess the Efficacy and Safety of Continuous Glucose Monitoring in Young Adults 14-<25 with Type 1 Diabetes” (CITY).^19^ By design, the study recruited adolescents and young adults with T1D (duration >12 months) exhibiting poorer glycemic control (HbA1c 7.5-<11.0%), most likely to benefit from CGM usage.^16^ The study was conducted at 14 endocrinology practices in the United States, where participants were randomly assigned to either CGM (Dexcom G5) or regular finger-prick glucose meter monitoring. The randomized trial aimed to determine the effect of CGM on glycemic control primarily measured through HbA1c. The CGM users were compared with the control group using HbA1c levels after six months of usage. After six months, all study participants were provided with CGM devices and HbA1c tracked for a further six months. The data were collected from January 2018 to November 2019, before being made publicly available in March 2020.

**Figure 1.**
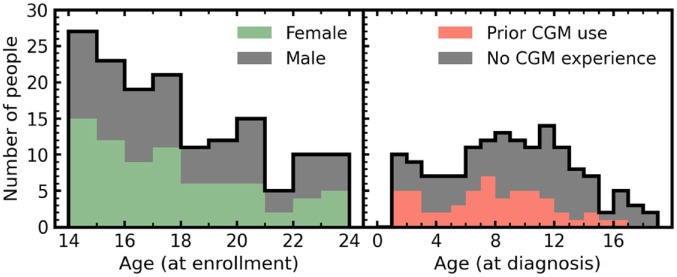
Distributions of age at enrollment (left) and age at original T1D diagnosis (right) for the 153 people with CGM data used in this study. Both distributions are stacked to show the breakdown of gender (left) and prior CGM usage (right) for the individuals. Abbreviations: CGM, continuous glucose monitoring; T1D, type 1 diabetes.

We make use of CGM data from 153 people living with T1D in the CITY study, where users were provided CGM devices for 6 to 12 months, totaling more than 28 000 days of usage data. In Figure 1, we show the breakdown of age (both at enrollment and at original diagnosis) for the 153 individuals. In addition to CGM data, basic screening information and the most recently recorded HbA1c test result were used to generate predictions. All participants had scheduled in-clinic visits throughout the study with HbA1c being routinely collected every 13 weeks after the baseline taken at screening.

### Features

To utilize CGM data for hypoglycemic and hyperglycemic predictions, we generate a total of 30 features which summarize a young adult’s CGM data on different timescales. Glucose control is summarized on short-term (one hour), medium-term (one day), and long-term (one week) baselines prior to the current CGM reading. This is combined with six features that characterize basic information. A complete description of all generated features is given in Table 1. Features are generated at the point of each unique CGM reading. Features are only used in modeling if the CGM device has been used for ≥80% for the prior week.

**Table 1.** Summary of Input Features Used by the Models to Make Predictions. A Subset of Features Are Computed for Various Time Ranges (ie, One Hour, One Day, One Week) and Considered as Independent Features.

Feature	Description	Time period (s)
Current reading	Most recent CGM glucose reading	N/A
Time of day	Hour (0-24) at which reading was reported	N/A
Day of week	Day on which reading was reported	N/A
Gender		N/A
Diagnosis age	Age at initial diagnosis of T1D	N/A
Prior use of CGM	Whether person with T1D had previous experience of using CGM before the study	N/A
Age	Age at study commencement	N/A
Years since original diagnosis	Year since initial diagnosis of T1D	N/A
Most recent HbA1c	Most recent recorded test result of HbA1c	N/A
Device usage fraction	Fraction of time (as specified by the time period) of which the CGM device was used	(One hour, one day, one week)
Fraction of time high	Fraction of time (as specified by the time period) of which CGM readings were above 270 mg/dL	(One hour, one day, one week)
Fraction of time low	Fraction of time (as specified by the time period) of which CGM readings were below 70 mg/dL	(One hour, one day, one week)
Average	Mean of glucose readings over specified time period	(One hour, one day, one week)
Standard deviation	Standard deviation of glucose readings over specified time period	(One hour, one day, one week)
Largest increase between readings	Largest increase in glucose level between consecutive readings within specified time period	(One hour, one day, one week)
Largest decrease between readings	Largest decrease in glucose level between consecutive readings within specified time period	(One hour, one day, one week)
Maximum number of consecutive increases	Most consecutive readings where glucose levels increase over defined time period	(One hour, one day, one week)
Maximum number of consecutive decreases	Most consecutive readings where glucose levels decrease over defined time period	(One hour, one day, one week)

Abbreviations: CGM, continuous glucose monitoring; T1D, type 1 diabetes; HbA1c, glycosylated hemoglobin or hemoglobin A1c.

### Targets

**Figure 2.**
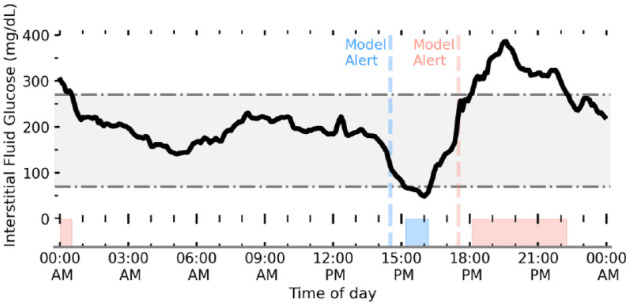
Schematic of interstitial fluid glucose levels (black line) for a young adult with T1D tracked by CGM. The gray-shaded region shows the desired range to keep glucose levels between 70 mg/dL < G < 270 mg/dL. Our algorithm aims to predict (ahead-of-time) when a person with T1D will go below (hypoglycemia) and above (hyperglycemia) this range. Regions of low and high glucose are shaded blue and red, respectively, with the corresponding first prediction event horizon (ie, when our model first made a positive prediction of hypo/hyper) shown by the dashed line. Abbreviations: T1D, type 1 diabetes; CGM, continuous glucose monitoring.

To generate targets for our model predictions, we generate two binary variables referring to hypoglycemic (<70 mg/dL) and hyperglycemic (>270 mg/dL) events. A feature set is generated for each unique CGM reading, at which point we check if the CGM user’s glucose level falls within these regions (for readings ≥1) in the following 60 minutes (ie, positive prediction). Our models therefore make predictions of hypoglycemia or hyperglycemia up to a maximum of 60 minutes ahead-of-time. Our average prediction horizon is 44 minutes. Glucose readings already within the hypoglycemic or hyperglycemic regions are removed from the modeling data set to avoid artificially boosting model performance metrics. Figure 2 shows a schematic of interstitial fluid glucose levels through a given day, regions of hypoglycemia and hyperglycemia, and timestamps of model predictions prior (ie, target).

### Modeling

To determine the added value of machine learning, we evaluate a baseline heuristic model, a logistic regression model, and a gradient boosted tree–based model for both hypoglycemia and hyperglycemia predictions. Our baseline heuristic model is equivalent to a glucose threshold alert (ie, predicting hypoglycemia and hyperglycemia within the next 60 minutes if interstitial fluid glucose levels fall below 110 mg/dL or go above 240 mg/dL, respectively).

Our logistic regression model is aimed to emulate basic CGM alerts which extrapolate linear trends along with thresholds to make hypoglycemia or hyperglycemia predictions. Logistic regression, despite its name, is a linear model which aims to classify binary outcomes through probabilities estimated by the logistic function. We implement our logistic regression model using Scikit-learn, optimized for ≤300 iterations using a L2 penalty with class weights inversely proportional to class frequencies.^30^ Finally, we make use of the XGBoost framework to implement a tree-based machine learning algorithm.^31^ XGBoost makes use of an ensemble of weak learners (ie, small trees) that are trained stagewise through gradient boosting. This reduces overfitting while preserving or lowering variance in the prediction error,^32^ which frequently leads to gradient boosted trees outperforming other tree-based methods. In addition, XGBoost naturally deals with continuous, binary/discrete, and missing data consistently, all of which are represented in our data set. Model hyperparameters for our XGBoost models were selected using fivefold cross-validation of the training set only using a sampler (Tree-structured Parzen Estimator) implemented with the Optuna library.^33^ In Supplemental Table 1, we include the complete set of tuned hyperparameters along with the corresponding search ranges.

We separate our CGM data into a hold-out test set (25%) and a training set (75%). An individual’s complete set of CGM data is allocated to only of the training or test set so that there is no data leakage or overfitting when evaluating model performance. Regardless, we note that randomly separating data (so that an individual’s CGM data can be split between both train and test sets) only marginally improves model performance.

Our supervised models (ie, logistic regression and XGBoost) learn from the training set, and all models are evaluated using the same test sample. Since we only filter data based on an individual’s average usage over the prior week (≥80% to be included in the feature set), there are rare occurrences where we have insufficient data to compute trends (eg, mean, standard deviation [SD], consecutive changes) and have missing feature values for these entries. This most commonly occurs when an individual replaces their sensor, meaning that there is a significant gap in the prior hour’s readings. To compare model performance fairly, we remove data with missing values from the test samples. We note, however, including entries with missing values for the XGBoost models (which can natively deal with missing values, unlike the logistic regression models) does not result in a significant performance decrease. Overall, model performance was evaluated using the area under the receiver operating curve (AUROC) and average precision, along with fixed measures of specificity and sensitivity.

### Model Explanability

Historically, machine learning algorithms are considered “black boxes” with little understanding of how predictions have been made. However, recent advances in *explanability* have led to individual predictions of tree-based algorithms being readily explainable.^34^

To attribute the relative importance of each feature in predicting both hypoglycemia and hyperglycemia risks for our XGBoost model, we make use of the TreeExplainer algorithm as implemented in the SHAP (SHapley Additive exPlanations) library.^34-36^ TreeExplainer efficiently calculates Shapley (SHAP) values,^37^ which aim to attribute payout (ie, the prize) between coalitional players of a game. In the context of machine learning, SHAP values amount to the marginal contribution (ie, change to the model prediction) of a feature among all possible coalitions (ie, combinations of features). Practically, this means that for every individual prediction (negative or positive), the relative importance of every feature can be evaluated.

There is a rich history of global interpretation for machine learning models which summarize the average overall importance of features on predictions as a whole.^38^ In a medical setting, however, tailored explanations for individuals are paramount, maximizing the ability to understand their own data and ensure every person is evaluated fairly.^39^ Shapley values are *locally accurate*, meaning that they can explain which features were relatively most important for an individual prediction (ie, a hypoglycemic or hyperglycemic event). In addition, Shapley values are consistent (the values add up to the actual prediction of the model) meaning they can also be used to check the global importance of a feature. Feature importance can therefore be checked periodically by averaging over a fixed time period. Practically, this means that for a CGM user over a given time period, the most important features leading to a prediction of hypoglycemia or hyperglycemia can be automatically evaluated. This gives immediate insight about an individual’s glucose control, and intuition about what may be increasing their risk. Presenting reliable predictions with intuitive explanations would enable users to be proactive in their control. Insightful control recommendations could empower users to feel closer to being on “autopilot” (ie, minimizing the cognitive load burden).

We choose to implement SHAP over other local explainer algorithms (eg, Lime)^40^ since SHAP offers mathematical guarantees of trustworthiness (local accuracy, missingness, and consistency) which adhere to strict medical governance guidelines,^34^ and offers consistency between local explanations meaning global importance can be computed as well.

## Results

### Model Evaluation

**Figure 3.**
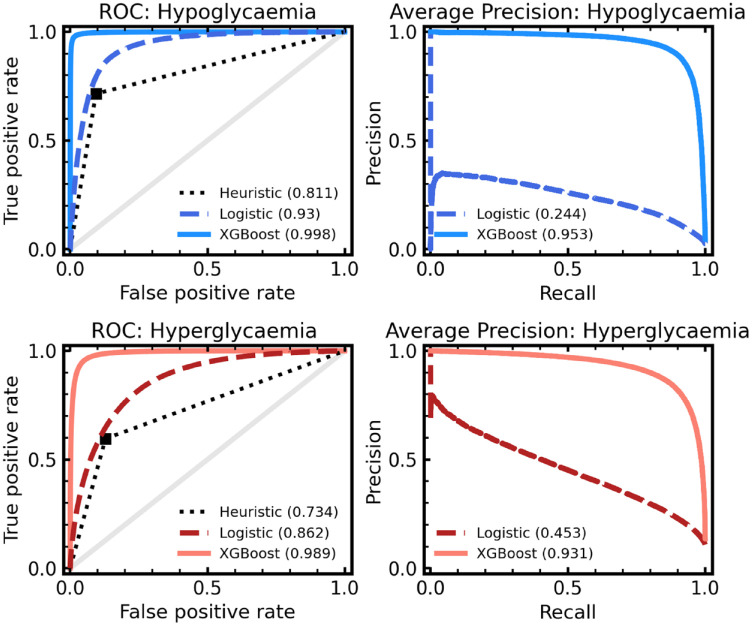
ROC (left) and average precision (right) for our models of hypoglycemia (blue; top row) and hyperglycemia (red; bottom row) predictions. In each panel, a XGBoost model (solid line) and a logistic regression model (dashed line) are given, and for ROC only are compared with a baseline heuristic (dotted line). A zero skill model is represented by the solid gray line also for the ROC panels. The total area under each curve (ie, AUROC score or average precision) is given in the brackets. Abbreviations: ROC, receiver operating curve; AUROC, area under the receiver operating curve.

In Figure 3, we compare the performance of our baseline heuristic model against the machine learning classifiers (ie, logistic regression and XGBoost). Performance is evaluated by the AUROC and average precision characteristics by comparing the model predictions of hypoglycemia (left) or hyperglycemia (right) up to 60 minutes ahead of time to the actual future readings. For hypoglycemia, the baseline model achieved an AUROC of 0.811, the logistic regression 0.930 (95% confidence interval [CI]: 0.929-0.931), and the XGBoost 0.998 (95% CI: 0.998-0.998) evaluated on our hold-out test set. In terms of average precision, the baseline model achieved 0.121, the logistic regression 0.244 (95% CI: 0.240-0.247), and XGBoost 0.953 (95% CI: 0.951-0.954). All CIs are estimated from bootstrapping (sampling with replacement) for 500 resamples per model.

We find a clear advantage in using XGBoost; however, the logistic regression model also performs reasonably. We note that despite its crudeness, our baseline heuristic model is still predictive, demonstrating the use of threshold-based alerts on CGM devices in forward planning. Regardless, a more powerful predictive model means a lower false-alarm rate can be achieved, while maintaining the safety of the predictions. Reducing alarm fatigue for CGM users is an important goal, and more skillful models help enable this. In Table 2, measures of model skill are given, including AUROC, average precision, sensitivity, and specificity. Sensitivity and specificity are evaluated from dichotomizing model predictions at probability *P* = .5. Again, we find a clear performance increase for our XGBoost model, in keeping with the high performance of decision tree–based methods^41^ and commercial hybrid loop systems.^42^

**Table 2.** Summary of Model Performance Metrics for Both Hypoglycemia and Hyperglycemia Predictions. A Baseline Heuristic, Logistic Regression, and an XGBoost Model Are Evaluated for Each Target. Summary Statistics (AUROC and Average Precision) Are Shown With 95% CI in Square Brackets. Sensitivity and Specificity Are Evaluated From Dichotomizing Model Predictions at Probability *P* = .5.

Model	AUROC	Average precision	Specificity (*P*_thres_ = .5)	Sensitivity (*P*_thres_ = .5)
**Hypoglycemia**				
Heuristic	0.811	0.121	.906	.716
Logistic regression	0.930 [0.929-0.931]	0.244 [0.240-0.247]	.827	.905
XGBoost	0.998 [0.998-0.998]	0.953 [0.951-0.954]	.994	.945
**Hyperglycemia**				
Heuristic	0.733	0.258	.872	.595
Logistic regression	0.862 [0.861-0.862]	0.453 [0.450-0.456]	.752	.817
XGBoost	0.989 [0.989-0.990]	0.931 [0.930-0.932]	.931	.970

Abbreviations: AUROC, area under the receiver operating curve; CI, confidence interval.

High performance is also seen for hyperglycemia, with the baseline model achieving an AUROC of 0.734, the logistic regression 0.862 (95% CI: 0.861-0.862), and XGBoost 0.989 (95% CI: 0.989-0.990). Average precision, sensitivity, and specificity demonstrate similar trends with XGBoost being the most skillful. For each modeling approach, we note that the model skill is lower for hyperglycemia prediction in comparison with hypoglycemia, suggesting prediction of lower glucose events is better suited to our modeling choices.

### Model Explanation

**Figure 4.**
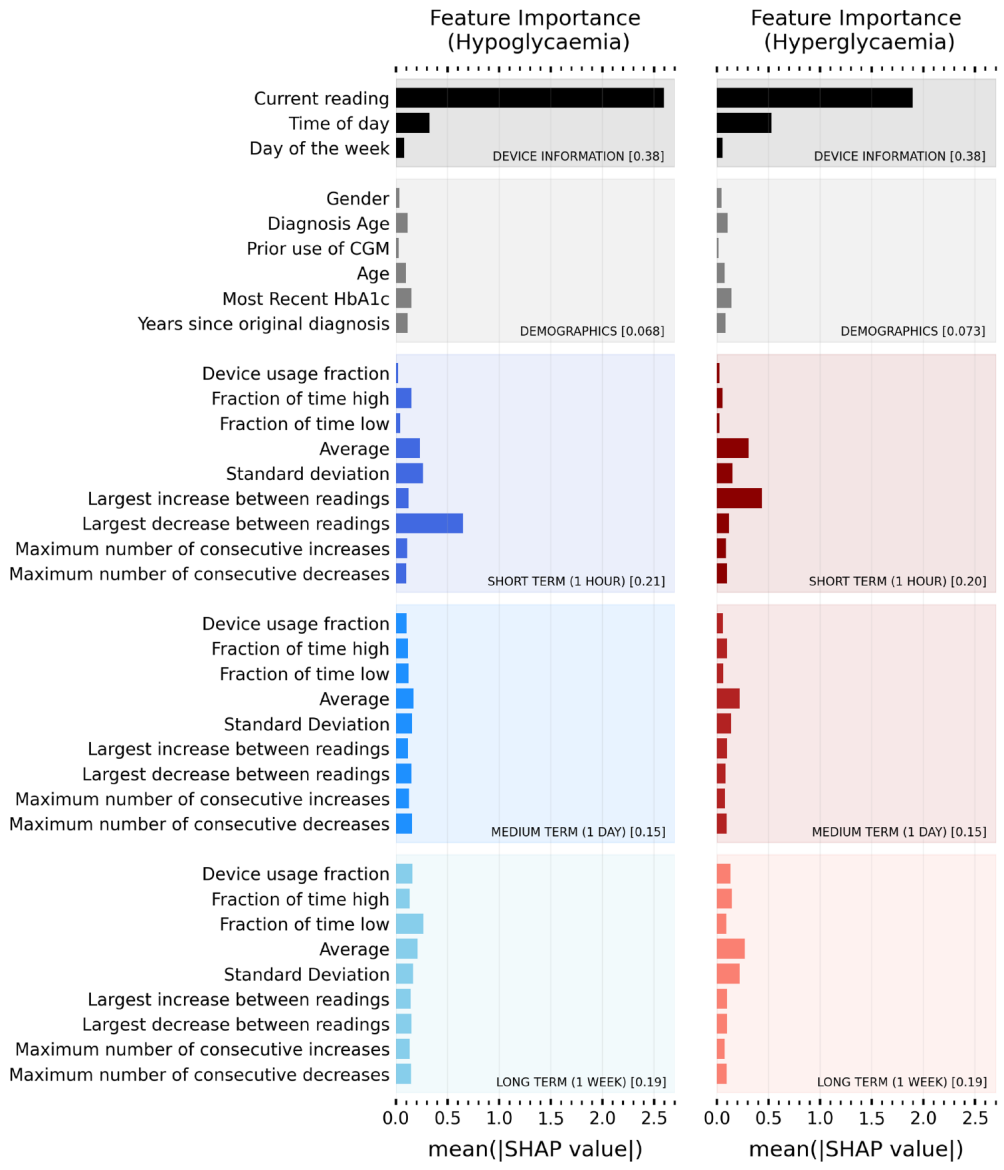
Overall importance ranking of input features for predicting hypo (left panel) and hyper (right panel) risk. Average (absolute) SHAP value for predictive features over all study participants. A higher value corresponds to a more important feature in decision-making. Features are grouped into categories (device information, demographics, short term [one hour], medium term [one day], long term [one week]). The fractional contribution (ie, sum over all features in that category) of a given category is given in the square brackets. Abbreviations: CGM, continuous glucose monitoring; SHAP, SHapley Additive exPlanations; HbA1c, glycosylated hemoglobin or hemoglobin A1c.

In addition to increased predictive power, the added value of machine learning models can be demonstrated through explanations. Using SHAP we can evaluate the relative importance of features for a given positive prediction of hypoglycemia or hyperglycemia. SHAP is applied post model construction and therefore has no negative implications for performance. Figure 4 shows the overall relative importance of every input feature for predicting hypoglycemic (left panel) and hyperglycemic (right panel) events. The relative importance of a feature is quantified by the absolute average SHAP value. Since SHAP values are consistent across predictions, they can be averaged for individual CGM users, across any time range, to provide immediate insight.

Here, we provide the average relative importance for all CGM users in the study, but this diagram is trivially made for individual users. Unsurprisingly, the user’s current glucose reading is most important for the model to make predictions of both hypoglycemia and hyperglycemia. Time of day is also important, providing insight into the sleep and eating, physical activity and stress level, and habits of the CGM user and their relationship with glycemic control. Sudden drops (or increases) in glucose are important for predicting hypoglycemia (hyperglycemia) as shown by the short-term largest decrease (increase) between readings. Interestingly, the long-term fraction of time low is found to be reasonably predictive of hypoglycemic events, providing immediate insight into certain user’s control habits.

### User Interface

**Figure 5.**
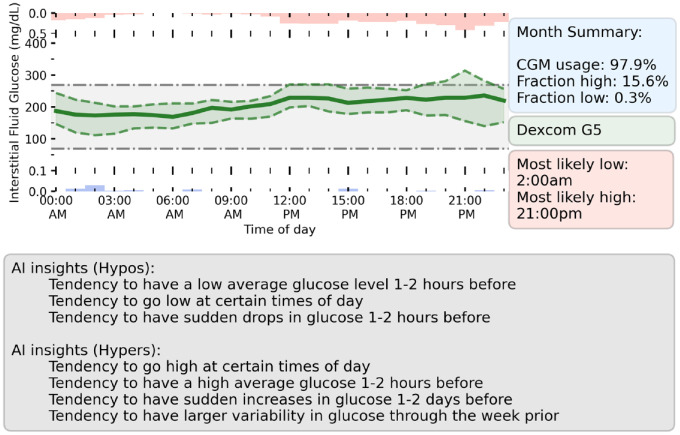
Prototype dashboard which summarizes the prior month of user’s CGM data and provides insights from explainable machine learning. (Upper left) Hourly average of glucose levels (solid green line) through the month. Upper and lower quartiles for each hour is shown by the green-shaded region. The hourly average for the fraction of time spent with high glucose (red, above axis) and low glucose (blue, below axis) is shown. (Upper right) Summary information about user’s CGM usage and glycemic control. (Bottom) Panel summarizing the AI control recommendations for the user over the past month. This was found by considering the most important features in the user’s CGM data for predicting hypoglycemia or hyperglycemia. Abbreviation: CGM, continuous glucose monitoring; AI, Artificial Intelligence.

Despite CGM providing a wealth of information to both users and clinicians, the sheer volume of data makes it hard to quickly draw conclusions about glycemic control. Quick summary metrics such as the fraction of time-in-range (eg, 70 mg/dL < G < 270 mg/dL) are the baseline for assessing control. By considering the most predictive model features that led to predictions of hypoglycemic or hyperglycemic events, we can draw further personalized insights into an individual’s glycemic control. In Figure 5, we present a prototype dashboard which summarizes a randomly selected user’s CGM data over a given month, along with potential insights derived from explainable machine learning. In addition to metrics such as time above or below range, we provide the user’s average glucose through the day, along with the most likely times for our model to predict hypoglycemia (red, above green line) or hyperglycemia (blue, below green line) for the individual. We select the top features for predicting both hypoglycemia and hyperglycemia for the user and summarize this information as control recommendations in the gray box. This provides a quick glance into the specifics of the user’s glycemic control, enabling the user to be better informed to avoid potential events in the future. One Artificial Intelligence (AI) insight (gray box) for this user is that they tend to go high at specific times of day. Looking at the fraction of time spent high on the dashboard through the day (red box and histogram), this peaks around 21:00 pm, hence the user should consider insulin dosages around their evening meal.

## Discussion

The key contributions of our work are as follows:

1. Machine learning models with state-of-the-art performance for predicting hypoglycemia (AUROC: 0.998) and hyperglycemia (AUROC: 0.989) up to 60 minutes in advance (44 minutes average event horizon). This performance is high relative to simple algorithms^43-45^ and comparable machine learning approaches.^23,46^
2. With careful feature engineering, we have demonstrated how machine learning explanations (SHAP) can be utilized to understand specifics about an individual’s control. SHAP also adds transparency to model predictions, aiding assurance that all individuals are evaluated fairly.
3. Provided a prototype dashboard to help young adults with T1D and clinicians make use of CGM data and the insight from machine learning explanations.

Technological advances represent a significant opportunity to help reduce self-care burden on an individual with T1D, and reduce the risk of health complications arising from poor glycemic control. In particular, for young adults, automated feedback from CGM may be an important tool for reducing risk, at times of transition (from pediatric to adult care units) and where glycemic control can be at a minimum.

Ahead-of-time machine learning predictions are of personal and clinical value as they give the CGM user more time to adjust self-care and reduce risk. Our tree-based model demonstrated a significant performance increase relative to threshold-based and linear models. This performance increase is vital for reducing alert burden on the user, since more certain predictions require less total alerts while maintaining safety of the device.

Despite the wealth of information provided by CGM devices, part of the problem is deriving quick insight that is useful for people with T1D, their family carers, and clinicians.^47,48^ Machine learning explanations can help summarize what specifics in an individual’s glycemic control led to increased risk of either hypoglycemia or hyperglycemia. Used in combination with directly derived metrics (eg, time-in-range), their utility can be in providing quick-glance–specific recommendations about how to reduce risk.

### Limitations

Limitations of this work include the reliance on the user to comply in using the CGM device. For our results, we only generate predictions when the user has used the device for 80% of the prior week. While predictions can still be generated with a lower usage compliance, this will inevitably decrease prediction performance, and care must be taken about when machine learning enhancement can be implemented safely. Furthermore, while current CGM devices are generally accurate, they are not infallible and considerations must be made for the safety of systems reliant on their accuracy.^49^

Another limitation of this study is the lack of insulin and carbohydrate data. Including this information could enable specific recommendations about insulin and carbohydrate dosages through the day. Including information tracked by smart watches, such as physical activity and stress levels, would not only improve predictions, but provide far more powerful intuitive recommendations. Having contextual information (eg, high stress levels or even self-reported event markers such as drinking, sickness, or exercise) would be critical for empathetic recommendations and reducing burden for the user.

Another limitation is the simplicity of our selected features to represent glycemic variability. Including additional metrics such as average daily risk range,^50^ %coefficient of variation,^51^ level 2 hypoglycemia (% of readings or time <54 mg/dL), level 2 hyperglycemia (% of readings or time >250 mg/dL),^52^ and low/high blood glucose index^53,54^ along with our baseline of standard deviation is likely to improve model performance. For example, reevaluating our XGBoost hypoglycemia model with the inclusion of %coefficient of variation (SD expressed as a percentage of the mean) and % of readings <54 mg/dL (over one hour, one day, and one week) we find an increase in average precision to 0.959 (up from 0.953) with AUROC remaining the same. However, including multiple measures of glycemic variability would confound our ability to clearly evaluate relative importance between feature types, and hence limit our ability to provide basic explanations. To maximize the utility of further predictive models, it will be important to include more complex features while preserving the ability to feedback specifics about an individual’s glycemic control.

In this work, we chose to train and test hypoglycemia and hyperglycemia models using data from all CGM users in our cohort. In practice, it may be more suitable to train *individual* models per CGM user, which may be better tailored to the individual. However, it would be more complex to make direct comparisons between relative feature importance for different CGM users, and hence left outside the scope of this article.

## Conclusion

We introduced a framework for high-performance prediction and explanation of hypoglycemia and hyperglycemia for young adults. Careful feature selection enables both accurate short-term risk prediction and intuitive feedback about an individual’s glucose control. The key benefit of adopting a machine learning framework lies in the ability to provide more accurate ahead-of-time predictions (in comparison with more simplistic-derived alerts), potentially reducing burden on the young adult potentially going through transition with their care practices. Combining these models with explanations enables both users and clinicians to gain immediate insight into an individual’s glycemic control, automatically highlighting what specific trends lead to increased risk.

## Supplemental Material

sj-docx-1-dst-10.1177_19322968221103561 – Supplemental material for Explainable Machine Learning for Real-Time Hypoglycemia and Hyperglycemia Prediction and Personalized Control Recommendations Supplemental material, sj-docx-1-dst-10.1177_19322968221103561 for Explainable Machine Learning for Real-Time Hypoglycemia and Hyperglycemia Prediction and Personalized Control Recommendations by Christopher Duckworth, Matthew J. Guy, Anitha Kumaran, Aisling Ann O’Kane, Amid Ayobi, Adriane Chapman, Paul Marshall and Michael Boniface in Journal of Diabetes Science and Technology

## References

[1] Inchiostro S Candido R Cavalot F. How can we monitor glycaemic variability in the clinical setting? Diabetes Obes Metab. 2013;15(suppl 2):13-16. 24034515 10.1111/dom.12142

[2] Leese GP Wang J Broomhall J , et al. Frequency of severe hypoglycemia requiring emergency treatment in type 1 and type 2 diabetes: a population-based study of health service resource use. Diabetes Care. 2003;26(4):1176-1180. 12663593 10.2337/diacare.26.4.1176

[3] Skrivarhaug T Bangstad HJ Stene LC Sandvik L Hanssen KF Joner G. Long-term mortality in a nationwide cohort of childhood-onset type 1 diabetic patients in Norway. Diabetologia. 2006;49(2):298-305. 16365724 10.1007/s00125-005-0082-6

[4] Musen G Jacobson AM Ryan CM , et al. Impact of diabetes and its treatment on cognitive function among adolescents who participated in the Diabetes Control and Complications Trial. Diabetes Care. 2008;31(10):1933-1938. 18606979 10.2337/dc08-0607 PMC2551630

[5] Lauretti E Li J-G Di Meco A Praticò D. Glucose deficit triggers tau pathology and synaptic dysfunction in a tauopathy mouse model. Transl Psychiatry. 2017;7(1):e1020. 28140402 10.1038/tp.2016.296 PMC5299397

[6] Emerging Risk Factors Collaboration. Diabetes mellitus, fasting blood glucose concentration, and risk of vascular disease: a collaborative meta-analysis of 102 prospective studies. Lancet. 2010;375(9733):2215-2222. 20609967 10.1016/S0140-6736(10)60484-9 PMC2904878

[7] Forbes JM Cooper ME. Mechanisms of diabetic complications. Physiol Rev. 2013;93(1):137-188. 23303908 10.1152/physrev.00045.2011

[8] Zhou B Lu Y Hajifathalian K , et al. Worldwide trends in diabetes since 1980: a pooled analysis of 751 population-based studies with 4.4 million participants. Lancet. 2016;387(10027):1513-1530. 27061677 10.1016/S0140-6736(16)00618-8 PMC5081106

[9] Borus JS Laffel L. Adherence challenges in the management of type 1 diabetes in adolescents: prevention and intervention. Curr Opin Pediatr. 2010;22(4):405-411. 20489639 10.1097/MOP.0b013e32833a46a7 PMC3159529

[10] Datye KA Moore DJ Russell WE Jaser SS. A review of adolescent adherence in type 1 diabetes and the untapped potential of diabetes providers to improve outcomes. Curr Diab Rep. 2015;15(8):51-59. 26084580 10.1007/s11892-015-0621-6 PMC4692366

[11] Clarke WL Gonder-Frederick A Snyder AL Cox DJ. Maternal fear of hypoglycemia in their children with insulin dependent diabetes mellitus. J Pediatr Endocrinol Metab. 1998;11(suppl 1):189-194. 9642659 10.1515/jpem.1998.11.s1.189

[12] Patton SR Dolan LM Henry R Powers SW. Fear of hypoglycemia in parents of young children with type 1 diabetes mellitus. J Clin Psychol Med Settings. 2008;15(3):252-259. 19104970 10.1007/s10880-008-9123-x PMC2737676

[13] Haugstvedt A Wentzel-Larsen T Graue M Søvik O Rokne B. Fear of hypoglycaemia in mothers and fathers of children with type 1 diabetes is associated with poor glycaemic control and parental emotional distress: a population-based study. Diabet Med. 2010;27(1):72-78. 20121892 10.1111/j.1464-5491.2009.02867.x

[14] Juvenile Diabetes Research Foundation Continuous Glucose Monitoring Study Group. Continuous glucose monitoring and intensive treatment of type 1 diabetes. N Engl J Med. 2008;359(14):1464-1476. 18779236 10.1056/NEJMoa0805017

[15] Juvenile Diabetes Research Foundation Continuous Glucose Monitoring Study Group. Effectiveness of continuous glucose monitoring in a clinical care environment: evidence from the Juvenile Diabetes Research Foundation continuous glucose monitoring (JDRF-CGM) trial. Diabetes Care. 2010;33(1):17-22. 19837791 10.2337/dc09-1502 PMC2797966

[16] Rodbard D. Continuous glucose monitoring: a review of successes, challenges, and opportunities. Diabetes Technol Ther. 2016;18(suppl 2):S3-S13. 10.1089/dia.2015.0417 PMC4717493 26784127

[17] Langendam M Luijf YM Hooft L Devries JH Mudde AH Scholten RJPM . Continuous glucose monitoring systems for type 1 diabetes mellitus. Cochrane Database of Syst Rev. 2012;1(1):CD008101. 10.1002/14651858.CD008101.pub2 PMC6486112 22258980

[18] Liebl A Henrichs HR Heinemann L , et al. Continuous glucose monitoring: evidence and consensus statement for clinical use. J Diabetes Sci Technol. 2013;7(2):500-519. 23567009 10.1177/193229681300700227 PMC3737652

[19] Laffel LM Kanapka LG Beck RW , et al. Effect of continuous glucose monitoring on glycemic control in adolescents and young adults with type 1 diabetes: a randomized clinical trial. JAMA. 2020;323(23):2388-2396. 32543683 10.1001/jama.2020.6940 PMC7298603

[20] Buckingham BA Bailey TS Christiansen M , et al. Evaluation of a predictive low-glucose management system in-clinic. Diabetes Technol Ther. 2017;19(5):288-292. 28221823 10.1089/dia.2016.0319

[21] Cichosz SL Frystyk J Hejlesen OK Tarnow L Fleischer J. A novel algorithm for prediction and detection of hypoglycemia based on continuous glucose monitoring and heart rate variability in patients with type 1 diabetes. J Diabetes Sci Technol. 2014;8(4):731-737. 24876412 10.1177/1932296814528838 PMC4764234

[22] van Doorn WPTM Foreman YD Schaper NC , et al. Machine learning-based glucose prediction with use of continuous glucose and physical activity monitoring data: the Maastricht Study. PLoS One. 2021;16(6):e0253125. 34166426 10.1371/journal.pone.0253125 PMC8224858

[23] Dave D DeSalvo DJ Haridas B , et al. Feature-based machine learning model for real-time hypoglycemia prediction. J Diabetes Sci Technol. 2021;15(4):842-855. 32476492 10.1177/1932296820922622 PMC8258517

[24] Jensen MH Dethlefsen C Vestergaard P Hejlesen O. Prediction of nocturnal hypoglycemia from continuous glucose monitoring data in people with type 1 diabetes: a proof-of-concept study. J Diabetes Sci Technol. 2020;14(2):250-256. 31390891 10.1177/1932296819868727 PMC7196854

[25] Pérez-Gandía C Facchinetti A Sparacino G , et al. Artificial neural network algorithm for online glucose prediction from continuous glucose monitoring. Diabetes Technol Ther. 2010;12(1):81-88. 20082589 10.1089/dia.2009.0076

[26] Howsmon D Bequette BW. Hypo-and hyperglycemic alarms: devices and algorithms. J Diabetes Sci Technol. 2015;9(5):1126-1137. 25931581 10.1177/1932296815583507 PMC4667339

[27] Gani A Gribok AV Lu Y Ward WK Vigersky RA Reifman J. Universal glucose models for predicting subcutaneous glucose concentration in humans. IEEE Trans Inf Technol Biomed. 2010;14(1):157-165. 19858035 10.1109/TITB.2009.2034141

[28] Vehí J Contreras I Oviedo S Biagi L Bertachi A. Prediction and prevention of hypoglycaemic events in type-1 diabetic patients using machine learning. Health Informatics J. 2020;26(1):703-718. 31195880 10.1177/1460458219850682

[29] Centers for Disease Control and Prevention (CDC). Type 1 Diabetes. 2021. Cited 2022. https://www.cdc.gov/diabetes/basics/what-is-type-1-diabetes.html.

[30] Pedregosa F Varoquaux G Gramfort A , et al. Scikit-learn: machine learning in Python. J Mach Learn Res. 2011;12:2825-2830.

[31] Chen T Guestrin C . XGBoost: a scalable tree boosting system. In: Proceedings of the 22nd ACM SIGKDD International Conference on Knowledge Discovery and Data Mining. San Francisco, CA: Association for Computing Machinery; 2016:785-794.

[32] Breiman L. Bias, variance, and arcing classifiers. Tech. rep. 460, Statistics Department, University of California, Berkeley; 1996.

[33] Akiba T Sano S Yanase T Ohta T Koyama M. Optuna: a next-generation hyperparameter optimization framework. In: Proceedings of the 25th ACM SIGKDD International Conference on Knowledge Discovery & Data Mining. 2019.

[34] Lundberg SM Erion G Chen H , et al. Explainable AI for trees: from local explanations to global understanding. arXiv preprint arXiv:1905.04610; 2019. 10.1038/s42256-019-0138-9 PMC7326367 32607472

[35] Lundberg SM Lee S-I. A unified approach to interpreting model predictions. In: Proceedings of the 31st International Conference on Neural Information Processing Systems. 2017.

[36] Lundberg SM Nair B Vavilala MS , et al. Explainable machine learning predictions to help anesthesiologists prevent hypoxemia during surgery.Nature Biomedical Engineering. 2018;2:749–760. 10.1038/s41551-018-0304-0 PMC6467492 31001455

[37] Shapley LS. 17. A value for n-person games. In: Contributions to the Theory of Games (AM-28). Vol II. Princeton, NJ: Princeton University Press; 2016.

[38] Kuhn M Johnson K. Applied Predictive Modeling. Vol 26. New York: Springer; 2013.

[39] Rajkomar A Hardt M Howell MD Corrado G Chin MH. Ensuring fairness in machine learning to advance health equity. Ann Intern Med. 2018;169(12):866-872. 30508424 10.7326/M18-1990 PMC6594166

[40] Ribeiro MT Singh S Guestrin C . “Why should I trust you?” Explaining the predictions of any classifier. In: Proceedings of the 22nd ACM SIGKDD International Conference on Knowledge Discovery and Data Mining. 2016.

[41] Dave D Erraguntla M Lawley M , et al. Improved low-glucose predictive alerts based on sustained hypoglycemia: model development and validation study. JMIR Diabetes. 2021;6(2):e26909. 33913816 10.2196/26909 PMC8120423

[42] Forlenza GP Ekhlaspour L Breton M , et al. Successful at-home use of the tandem control-IQ artificial pancreas system in young children during a randomized controlled trial. Diabetes Technol Ther. 2019;21(4):159-169. 30888835 10.1089/dia.2019.0011 PMC6909715

[43] Biester T Kordonouri O Holder M , et al. “Let the algorithm do the work”: reduction of hypoglycemia using sensor-augmented pump therapy with predictive insulin suspension (SmartGuard) in pediatric type 1 diabetes patients. Diabetes Technol Ther. 2017;19(3):173-182. 28099035 10.1089/dia.2016.0349 PMC5359639

[44] Vu L Kefayati S Idé T , et al. Predicting nocturnal hypoglycemia from continuous glucose monitoring data with extended prediction horizon. AMIA Annu Symp Proc. 2019;2019:874-882. 32308884 PMC7153099

[45] Kodama S Fujihara K Shiozaki H , et al. Ability of current machine learning algorithms to predict and detect hypoglycemia in patients with diabetes mellitus: meta-analysis. JMIR Diabetes. 2021;6(1):e22458. 33512324 10.2196/22458 PMC7880810

[46] Deng Y Lu L Aponte L , et al. Deep transfer learning and data augmentation improve glucose levels prediction in type 2 diabetes patients. NPJ Digit Med. 2021;4(1):1-13. 34262114 10.1038/s41746-021-00480-x PMC8280162

[47] Polonsky WH Fortmann AL. Impact of real-time CGM data sharing on quality of life in the caregivers of adults and children with type 1 diabetes. J Diabetes Sci Technol. 2022;16(1):97-105. 33322931 10.1177/1932296820978423 PMC8875067

[48] Polonsky WH Hessler D. What are the quality of life-related benefits and losses associated with real-time continuous glucose monitoring? a survey of current users. Diabetes Technol Ther. 2013;15(4):295-301. 23427866 10.1089/dia.2012.0298

[49] Schrangl P Reiterer F Heinemann L Freckmann G Del Re L . Limits to the evaluation of the accuracy of continuous glucose monitoring systems by clinical trials. Biosensors(Basel). 2018;8(2):50. 29783669 10.3390/bios8020050 PMC6023102

[50] Kovatchev BP Otto E Cox D Gonder-Frederick L Clarke W. Evaluation of a new measure of blood glucose variability in diabetes. Diabetes Care. 2006;29(11):2433-2438. 17065680 10.2337/dc06-1085

[51] Rodbard D. Glucose variability: a review of clinical applications and research developments. Diabetes Technol Ther. 2018;20(S2):S25-S215. 29916742 10.1089/dia.2018.0092

[52] Battelino T Danne T Bergenstal RM , et al. Clinical targets for continuous glucose monitoring data interpretation: recommendations from the international consensus on time in range. Diabetes Care. 2019;42(8):1593-1603. 31177185 10.2337/dci19-0028 PMC6973648

[53] Kovatchev BP Cox DJ Gonder-Frederick LA Clarke W. Symmetrization of the blood glucose measurement scale and its applications. Diabetes Care. 1997;20(11):1655-1658. 9353603 10.2337/diacare.20.11.1655

[54] Kovatchev BP Cox DJ Gonder-Frederick LA Young-Hyman D Schlundt D Clarke W. Assessment of risk for severe hypoglycemia among adults with IDDM: validation of the low blood glucose index. Diabetes Care. 1998;21(11):1870-1875. 9802735 10.2337/diacare.21.11.1870

