# Supplementary material for: Explainable Machine Learning for Real-Time Hypoglycemia and Hyperglycemia Prediction and Personalized Control Recommendations
Source: J Diabetes Sci Technol. 2022 Jun 13;18(1):113–23. doi: 10.1177/19322968221103561 (PMC10899844; doi:10.1177/19322968221103561)
Supplement: sj-docx-1-dst-10.1177_19322968221103561 – Supplemental material for Explainable Machine Learning for Real-Time Hypoglycemia and Hyperglycemia Prediction and Personalized Control Recommendations [file sj-docx-1-dst-10.1177_19322968221103561.docx]

| Hyperparameter | Value | Search Range |
| --- | --- | --- |
| n_estimators | 425 | 25-700 |
| max_depth | 15 | 1-15 |
| learning_rate | 0.057 | 0.001-1 (logarithmic steps) |
| subsample | 0.85 | 0.1-1 |
| colsample_bytree | 0.53 | 0.1-1 |
| max_delta_step | 5 | 0-5 |
| min_child_weight | 4 | 0-5 |
| gamma | 1 | 0-5 |
| scale_pos_weight | 23.96 (hypo)  9.31 (hyper) | 1-40 |
| alpha | 3 | 0-5 |
| lambda | 5 | 0-5 |

**Supplementary Table 1:** Summary of model hyperparameters selected through tuning. Both models for hypoglycaemia and hyperglycaemia were tuned independently and converged on similar values, other than scale_pos_weight. Here we show the hyperparameters found from hypoglycaemia tuning which were used for both model architectures, other than scale_pos_weight. A description of each hyperparameter can be found at: <https://xgboost.readthedocs.io/en/stable/parameter.html> .
